# Stress-Induced Reversion to Virulence of Infectious Pancreatic Necrosis Virus in Naïve Fry of Atlantic Salmon (*Salmo salar* L.)

**DOI:** 10.1371/journal.pone.0054656

**Published:** 2013-02-19

**Authors:** Koestan Gadan, Ane Sandtrø, Inderjit S. Marjara, Nina Santi, Hetron M. Munang'andu, Øystein Evensen

**Affiliations:** 1 Norwegian School of Veterinary Science, Oslo, Norway; 2 Aqua Gen AS, Trondheim, Norway; INRA, France

## Abstract

We have studied stress-induced reversion to virulence of infectious pancreatic necrosis virus (IPNV) in persistently infected Atlantic salmon (*Salmo salar* L.) fry. Naïve fry were persistently infected with a virulent strain (T_217_A_221_ of major structural virus protein 2, VP2) or a low virulent (T_217_T_221_) variant of IPNV. The fry were infected prior to immunocompetence as documented by lack of recombination activating gene-1, T-cell receptor and B-cell receptor mRNA expression at time of challenge. The fish were followed over 6 months and monitored monthly for presence of virus and viral genome mutations. No mutation was identified in the TA or TT group over the 6 months period post infection. Six months post infection TA and TT infected groups were subject to daily stress for 7 days and then sampled weekly for an additional period of 28 days post stress. Stress-responses were documented by down-regulation of mRNA expression of IFN-α1 and concomitant increase of replication levels of T_217_T_221_ infected fish at day 1 post stress. By 28 days post stress a T221A reversion was found in 3 of 6 fish in the T_217_T_221_ infected group. Sequencing of reverted isolates showed single nucleotide peaks on chromatograms for residue 221 for all three isolates and no mix of TA and TT strains. Replication fitness of reverted (TA) and non-reverted (TT) variants was studied *in vitro* under an antiviral state induced by recombinant IFN-α1. The T_217_A_221_ reverted variant replicated to levels 23-fold higher than the T_217_T_221_ strain in IFN-α1 treated cells. Finally, reverted TA strains were virulent when tested in an *in vivo* trial in susceptible salmon fry. In conclusion, these results indicate that stress plays a key role in viral replication *in vivo* and can facilitate conditions that will allow reversion from attenuated virus variants of IPNV.

## Introduction

Infectious pancreatic necrosis virus (IPNV) is the causative agent of the infectious pancreatic necrosis (IPN) in salmonid fish, belongs to the family *Birnaviridae* and is the type strain of the genus Aquabirnaviruses [1]. IPN was previously regarded as a disease mainly of first-feeding fry, but the disease situation has changed over the past two decades, and outbreaks amongst post-smolt Atlantic salmon (*Salmo salar* L.) 6–10 weeks following transfer to sea-water have become a major threat to the economy of the fish farming industry [1], [2]. Transfer of young salmon to salt water is a particularly stressful stage in the production cycle, as smoltification involves a complex change in physiology, morphology, biochemistry and behaviour; preparing the fish for the transition from fresh water to marine life. IPN in post-smolts after sea transfer is considered a stress-mediated reactivation of an asymptomatic IPNV-infection as most IPNV infected fish become life-long carriers of the virus [2], [3]. Carrier fish shed virus in their faeces, however, titres fluctuate over time and increase during periods of stress [2]. IPNV is found in macrophage populations in the hematopoietic tissue of the kidney of persistently infected fish and IPNV can multiply in adherent leucocytes isolated from carriers, although it does not produce lytic infections [3]. Viruses residing in leucocytes can alter their function, for instance by disturbing the release of cytokines, antibodies or other molecules made by immune cells [4]. There are indications of reduced immune response in leucocytes isolated from carrier fish, and of increased virus replication upon stimulation of resting leucocytes [5]. There is no correlation between the presence of virus and the level of anti-IPNV antibodies in persistently infected fish [6], [7]. Strong Mx induction is observed in acute IPNV infection in post-smolt, but not in asymptomatic carriers, although the latter have the ability to respond with an Mx expression in response to poly I:C injection [6]. Further, epidemiological studies have identified transport stress as a risk factor for IPN outbreaks [7], [8] which aligns with the observation that IPN can be induced in covertly infected post-smolts by exposure to environmental stress under experimental conditions [9]. This supports the general notion that IPN outbreaks in sea water result from reactivation of a persistent infection.

The mortality of acute IPN outbreaks varies considerably, partly due to strain variation in virulence [10]. Amino acid residues 217, 221 and 247 were identified as important for virulence in a previous study of field strains of IPNV [10]. Later studies have shown that positions 217 and 221 are key in determining the virulence of serotype Sp strains [11]. Virulent strains have a combination of Thr and Ala in positions 217 and 221, respectively (T_217_A_221_) while strains of intermediate virulence carry P_217_A_221_. Strains with a T_217_T_221_ and P_217_T_221_ combination are avirulent [11]. Residues 217 and 221 of VP2 also influence the *in vitro* growth characteristics of IPNV Sp strains. In particular, an A221T substitution is involved in adaptation to CHSE-214 cells [10]. Attenuated viruses replicate faster and produce larger plaques in CHSE cells. IPNV causes persistent infection [12], [13] and in one previous study we showed that attenuated virus variants (T_217_T_221_) can occur late during infection in fish originally infected with a virulent strain (T_217_A_221_) [14]. In yet another study we showed that virulent strains (T_217_A_221_) do not establish a persistent infection as efficiently as avirulent strains (T_217_T_221_) [11]. Indeed, VP2 residue 221 seems to be a “hot spot” for adaptive mutation of the IPNV Sp genome, singularly affecting the virulence and persistence characteristics of the virus *in vivo*, as well as the growth characteristics in cell culture. In light of these observations, one issue that remains unaddressed in the literature is the possibility for reversion to virulence *in vivo* for IPNV strains.

On this basis we established a persistent infection with IPNV in fry of Atlantic salmon using one high (T_217_A_221_) and one low virulent (T_217_T_221_) strain. Naïve fry were infected prior to immunocompetence (0.15 g size). The fish were followed for 6 months post challenge and then exposed to acute stress over a period of 1 week. We found IFN-α1 and Mx gene expression levels were down-regulated and the replication level in T_217_T_221_ infected fish was up 8-fold at day 1 after stress and by 28 days post stress we found a reversion to a virulent wild-type (T_217_A_221_). The reverted variants showed higher replication levels at individual fish level. When tested for virulence in vivo, the reverted variants had attained their virulence profile. Further to this, we found that the T_217_A_221_ variant replicated under high IFN-α1 and Mx expression levels *in vivo.* And more so, *in vitro* the wild-type showed higher resistance to pre-treatment of cell cultures with rIFN-α1 in contrast to the less fit T_217_T_221_ strain where virus replication was completely blocked.

## Materials and Methods

### Cells used for culturing

Rainbow trout gonad (RTG-2) cells (ATCC CCL-55) and Chinook salmon embryo (CHSE-214) cells (ATCC CRL-1681) were grown at 20°C in L-15 medium (Sigma Aldrich) supplemented with 10% fetal bovine serum (FBS, Medprobe), 1% L-glutamin (Sigma Aldrich), and 50 μg ml^−1^ gentamicin (Sigma Aldrich). The TO cell line (macrophage cell line), originating from salmon head kidney leukocytes [15] were grown at 20°C in HMEM (Eagle's MEM with Hanks' BSS) supplemented with L-glutamine, MEM non-essential amino acids, gentamicin sulphate and 10% fetal bovine serum (FBS).

### Construction of cDNA clones

Generation of full-length cDNA clones of the entire coding and non-coding regions of NVI-015 RNA segment A and B was performed according to procedures described by Yao and Vakharia [16]. The recombinant IPNV Sp strains rNVI-15TA was generated as described in previous studies [10], [11]. Briefly, combining transcripts of pUC19NVI15A plus pU19NVI115B resulted in the recovery of the viral progeny designated as rNVI-15TA which has similar residues (T_217_A_221_) to the parent IPNV strain NVI-015. Once the genetically engineered virus strain was made, it was propagated on RTG-2 monolayers and the supernatants were harvested following low centrifugation and sterile filtration (0.22 μm). RNA extraction using the QIAamp viral mini kit (Qiagen) was carried out following the manufacturer's recommendation. Complete nucleotide sequences of segment A and B were determined as before [10]. The chromatograms were analyzed to ensure that the generated clones had the correct residues at positions 217, 221 and 247.

### Plaque purification assay

To generate an additional mutant virus in residue 221 of VP2, we took advantage of previously observed attenuation characteristics of the (T_217_A_221_) strains of IPNV where attenuation is seen in CHSE-214 following passage in culture [11], [14]. In brief, progeny viruses designated rNVI-15-TT representing one amino acid substitution of A221T of the parent strain rNVI-15-TA, were obtained after the recombinant strain and passaged on CHSE-214 cells. Mutations are seen already at 3–4 passages in culture and the virus passage up to the tenth passage, after which the isolate was plaque purified by inoculating RTG-2 monolayers on six well plates with 10-fold dilution (10^−3^ to 10^−8^) of cell culture supernatants. After 1 hr adsorption at room temperature the inoculum was removed and the cells were overlaid with 0.8% SeaPlaque Agarose (BioWhittaker) in L-15 medium containing 5% FBS and 1% L-glutamine. The cells were incubated at 15°C for 4 days and plaques formed by cytopathic effect (CPE) were removed from the wells using a punch biopsy equipment. Fifteen plaques were subsequently inoculated on RTG-2 monolayers and incubated at 15°C until full CPE was observed. The supernatant was harvested after low centrifugation and sterile filtration (0.22 μm). RNA was extracted using the QIAamp Viral RNA mini kit following the manufacturer's recommendations. Complete nucleotide sequences of segment A and B of each virus was determined as described before [10], [14], [17]. The chromatographs were checked to ensure a “clean” ACC codon encoding T_221_ was used in the study. No other mutations were found in the entire genome of the virus. Prior to challenge the isolates were propagated by one passage in RTG-2 cells. The cell culture supernatant was obtained after a brief centrifugation, and the infectious titer was determined by end point dilution on RTG-2 cells grown in 96 well plates. Fifty μl of 10-fold dilutions (10^−1^ to 10^−8^) of cell culture supernatants were inoculated in six parallel wells per dilution. The TCID_50_ was estimated by the method of Kärber [18].

### Establishing persistent infection of Atlantic salmon fry

The challenge was conducted at VESO Vikan's research facility, Namsos, Norway. A total of 770 Atlantic salmon (*Salmo salar* L.) fry of the AquaGen strain hatched at the VESO Vikan hatchery were included in the experiment at the time when the fry started to feed (Micro 015, Ewos). At arrival to the research facilities, 20 fish were sampled for measurement of average weight (0.15 g). The rest of the fish were divided into 3 tanks, each of 250 fry. After an acclimatization period of one week, the fry were starved one day before challenge. Fish were challenged by immersion with IPNV at a dose of 5×10^4^ TCID_50_/ml in a total volume of 4 liters per tank. One tank was challenged with rNVI-15TA (TA group), one tank was challenged with rNVI-15TT (TT), and one control (ctlr) tank was mock-infected by adding cell culture medium. The water was aerated during the challenge. After a period of 3 hours the water volume was reduced to 2 liters and normal flow was resumed. Mortality was recorded on a daily basis, and dead fish were collected each day and frozen at −70°C. Sampling of ten fish from each tank was performed at ten days post challenge, and after this first sampling, five fish were sampled from each tank once a month, up until 6 months post challenge.

### Stress challenge of IPNV carrier fish and uninfected controls

At six months post challenge, the remaining fish in each of the tanks were divided into two parallels; A and B; each parallel including 3 tanks and with approximately 50 fish in each tank. Fish in tanks 1B (TA-group), 2B (TT-group) and 3B (uninfected controls) were subject to three stressful events during a period of 7 days. The stress was imposed by reducing water level to approximately ½ of the normal water level. In addition, fish were chased for 15 minutes with a net, at a moderate speed. Fish in parallels 1A, 2A, and 3A (corresponding to the same groups as above) were not subject to any stress treatment (non-stressed).

Before splitting the fish in two parallel tanks, 5 fish were sampled from each group and frozen at−70°C, kidney was dissected from six fish in each group and stored in RNAlater® (Qiagen). After the stress treatment, samples were collected once per week for the four following weeks (7 days apart, days 1–28 post stress). At these samplings, twelve fish were collected from each group and six of them were frozen at −70°C while the kidney was dissected from the other six fish and stored in RNAlater®.

### Virus re-isolation from persistently infected fish

Fry samples stored at −70°C were homogenized in phosphate buffered saline (PBS) (1∶5, weight/volume) using a stomacher. 100 µl of this homogenate was transferred to RLT buffer containing 2-mercaptoethanol (RNeasy Mini kit, Qiagen) and stored at −70°C. The rest of the homogenate was diluted 1∶2 in L-15 medium supplemented with15 L-glutamine and 50 µg ml^−1^ (without FBS). After low centrifuging for 10 minutes, samples were inoculated onto RTG-2 cells grown in 24 well plates in final dilutions of 1 and 0.1%, and incubated for a week at 15°C. The cell culture medium after first passage was used to infect new monolayers followed by incubation for another week. Samples were considered negative when no CPE was observed after the second passage. RNA was isolated from fish homogenates for all negative samples using the RNeasy Mini kit (Qiagen) in accordance with the supplier's protocol. Qiagen's OneStep RT-PCR kit was used according to the manufacturer's instructions, with 0.5 μg RNA and 15 pmol each of primers IPNVF and IPNVR (Table 1) in a total reaction volume of 25 μl. The cycling conditions were 60°C for 30 min., 95°C for 15 min., followed by 45 cycles at 94°C for 45 s, 57°C for 45 s, 72°C for 1 min., and finally 72°C for 10 min. The PCR products were visualized by agarose gel electrophoresis. Three controls were included for each RT-PCR run: one positive and one negative tissue sample, and one negative control in which water was substituted for RNA.

**Table 1:** Primer used by RT-PCR.

Gene	Name	Direction	Sequence (5–3)	Length of amplicon(bp)	Annealing temp.
Elongation factor 1 alfa (Elgfα)	Elgf1α	Fwd	GCTGTGCGTGACATGAGG	88	60
		Rev	ACTTTGTGACCTTGCCGC		
Interferon alfa IFNa-1	IFNα-1	Fwd	TGGGAGGAGATATCACAAAGC	163	60
		Rev	TCCCAGGTGACAGATTTCAT		
Mx protein	Mx	Fwd	TGCAACCACAGAGGCTTTGAA	78	60
		Rev	GGCTTGGTCAGGATGCCTAAT		
IgM heavy chain membrane bound form IgM	IgM	Fwd	TCTGGGTTGCATTGCCACTG	121	60
		Rev	GTAGCTTCCACTGGTTTGGAC		
Recombination activating gene-1	RAG-1	Fwd	CCTAACACCTCTAGGCTTGAC	103	60
		Rev	GCTTCCCTGTTTACTCGC		
T cell receptor alpha chain	TCAC	Fwd	GCCTGGCTACAGATTTCAGC	107	66
		Rev	GGCAACCTGGCTGTAGTAAGC		
VP1	LVP1mPCR507	Fwd	CTGGTCCAGAAACCCTAAGAC	506	60
	RVP1mPCR1014	Rev	GTGTGTATCTCTCCCCTTTTGG		
RT-PCR primers					
Infectious pancreatic necrosis virus, A segment	IPNV1F	Fwd	ATCTGCGGAGTAGACATCAAAG	223	59
	IPNV2R	Rev	TGCAGTTCTTCGTCCATCCC		
Infectious pancreatic necrosis virus, A segment	A-Sp500F	Fwd	CAAGGATGGTATTCACCG	1189	59
	A-Sp1689R	Rev	AGCCTGTTCTTGAGGGCTC		

This table shows primer sequences for genes tested by quantitivate real-time RT-PCR for mRNA expression. The primers used for RT-PCR for detection of IPNV genome in kidney are given in the two last rows of the table.

### Sequencing

RNA was isolated from second passage by the Viral kit (Qiagen) and RT-PCR was performed to amplify a 400-bp IPNV-specific DNA fragment using Qiagen's OneStep RT-PCR kit according to the manufacturer's instructions, with 0,5 μg RNA and 15 pmol each of primers ASP500F and ASP1689R (Table 1) in a total reaction volume of 25 μl. The cycling conditions were 50°C for 30 min., 95°C for 15 min., followed by 40 cycles at 94°C for 45 s, 57°C for 45 s, 72°C for 2 min.15 s, and finally 72°C for 10 min. The PCR products were visualized by agarose gel electrophoresis. To purify the PCR-product fragments from agarose gel, the Quantum Prep Freeze N Squeeze DNA Gel Extraction Spin Column (BIO-RAD) was used according to the manufacturer's instructions. The recovered PCR-product was sequenced by a commercial sequencing service (Eurofins MWG operon) using primer ASp500F (Table 1). The sequence data were analyzed using PC/Gene (Intelligenetics) software.

### Expression of immune-related genes

To assess the stress effect on the immune response, real-time RT-PCR was carried out on kidney samples for a defined set of genes that included IFN-α1. mRNA expression of RAG-1, IgM, and TcR was evaluated at time of challenge (0.2 g size) and at 1 g and 2 g size. Elongation factor 1α was used as the reference genes for all gene tested. Expression of the viral polymerase (VP1) was used to document and assess IPNV replication at different time points (see below). In small fry (<1 g) whole fish were examined as it is impossible to dissect kidney tissue at this size. For larger fish (>1 g) kidney tissue were sampled on RNAlater and homogenized by metal beads (2.0 μm) in 700 RTL buffer containing 2-mercaptoethanol by mix-miller. RNA was isolated from all samples using the RNeasy Mini kit in accordance with the supplier's protocol (Qiagen).

The RNA concentration was measured using a ND-1000 Spectrophotometer (NanoDrop Technologies, Wilmington, DE, USA). 600 ng of total RNA was used for the production of cDNA, utilizing the Superscript III Reverse Transcriptase (Invitrogen) according to protocol for random hexamer primed cDNA synthesis. The samples were denatured at 65°C for 5 min, before the remaining components were added. The RT-step was performed at 50°C for 1 h, and finally the reaction was terminated by heating to 70°C for 15 min. Real-time PCR was run on the LightCycler 2.0 instrument using LightCycler®FastStart DNA Master PLUS SYBR Green I (Roche Diagnostics).

All real-time PCR runs were performed in duplicate and each real-time PCR master mix contained 5 μl water, 0.5 μl of each primer, 2 μl enzyme mix and 2 μl template. PCR conditions comprised of an initial incubation at 95°C for 10 min to activate hot-start polymerase followed by 40 cycles of 95°C for 10 s, annealing for 10 s and extension at 72°C. Primer sequences and other experimental conditions used for real-time PCR amplification are shown in Table 1. Melting curve analysis was performed to assess the specificity of PCR products, and all products were additionally tested by agarose gel electrophoresis to confirm the correct size of each product. Data analyses were performed using the LightCycler software version 4.0. The crossing points (Cp) was determined by use of the maximum second derivative function on the LightCycler® Software, and analyses of relative gene-expression, (elongation factor-1α; EGF-1α), were done with efficiency correction using external standards.

### Circulating antibodies

96-well microtiter plates were coated with 0.1 ml per well of a polyclonal rabbit anti-IPNV [19] onto 96 micro-titer plates (Immunoplates, Nunc Maxisorb, Denmark) at a dilution of 1∶5000. The coated plates were incubated overnight at 4°C. This was followed by three washing steps using 0.3 ml PBS/T (0.05%) per well. 0.2 ml of 5% dry milk in PBS/T was added to each well and the plates were left for incubation at room temperature for two hours. After washing, 0.1 ml of plaque purified TA variant of IPNV was added to each well. The virus was grown on RTG-2 cells, harvested when full CPE was observed, sterile filtered (0.22 μm) and diluted to a concentration of 1.0×10^5^TCID_50_/ml in 1% dry milk. After washing 0.l ml of the diluted plasma controls, blank (1% dry milk PBS/T), IPNV positive control sera and dilutions of the test sera were added to the wells on the plate. The test sera were diluted at 1∶50. Six wells for each control were used per plate. The plates were incubated at 4°C overnight. After washing 0.1 ml of a mouse monoclonal (IgG) anti-rainbow trout IgM [19] diluted at 1∶5000 in 1% dry milk was added to each well. The plates were left for incubation for 2 hrs at room temperature. This was followed by washing and adding 0.1 ml of horseradish peroxidase (HRP) conjugated anti-mouse IgG (Bio-Rad, USA) diluted 1∶5000 in 1% dry milk. After incubation and washing, 0.1 ml substrate solution (O-Phenylenediamine dihydrochloride tablets produced by DAKO, dissolved in distilled water mixed with 30% H_2_O_2_) was added to each well. Color development was stopped by adding 0.05 ml H_2_SO_4_ per plate. Results were read by using a spectrophotometer ELISA reader (TECAN, Genios) at a wavelength of 492 nm.

### Protein concentration quantification

The Quick start Bradford protein Assay and 1X dye bovine serum albumin (BSA) were used to determine the concentration of serum samples according to the manufacturer's instructions (Bio-RAD). Samples were diluted 10 and 20 folds to measure the protein concentration.

### Virulence testing of in vivo reverted strains

The reverted T_217_A_22_ isolates (3 isolates) and one isolate of the non-reverted variant, T_217_T_221_, all reisolated from fish at 7 months post challenge, were tested for their *in vivo* virulence in Atlantic salmon fry (0.5–0.8 g size). The titers were determined as described above. A total of 1500 fish of wild salmon fry originating from the river Rauma in Norway were used in the experiment. The eggs came from brood fish kept in the living gene bank (Haukvik) for more than 10 years. The eggs were hatched at VESO Vikan's hatchery, and transferred to the research station when ready for start-feeding. Fish were divided into 15 tanks each of 100 fry in triplicates tanks (T221T) 1×10^5^ TCID_50_/ml, (T221A**_1_**) 1×10^4^ TCID_50_/ml, (T221A**_2_**), 2×10^5^ TCID_50_/ml, and (T221A**_3_**) 1×10^5^ TCID_50_/ml. After one week of acclimatization the fry were starved one day prior to challenge, performed by administering a dose as mentioned above in a total volume of 2 liters per tank. There was no water exchange for 3 hours (oxygenation directly into the tanks) after which normal water flow resumed. All challenge materials were diluted to a total volume of 10 ml in cell culture medium. In the control tanks only cell culture media was added. Mortality was recorded on a daily basis and dead fish were collected each day and frozen at −70°C. They were analyzed by re-isolation of virus and sequenced using the methods described above.

### In vitro replication fitness

TO cells were seeded in 24-well plates and cultured until confluent. Cells were treated with 2.5 µg/ml rIFN-α1 and left for 24 h after which parallel wells were infected with rNVI-015-TA and rNVI-015-TT IPNV strains at MOI 1. Parallel TO cell cultures not pretreated with rIFN-α1 and infected with the same recombinant virus strains were included as controls. At 8, 24, 36, 48, and 60 h post infection, infected wells were harvested and virus replication was assessed by real-time RT-PCR using VP1 and VP2 specific primers. The data was expressed as the mean fold change in gene expression ± standard error of different dilutions of interferon treated groups relative to non-treated control groups after normalization with β-actin.

### Modeling

Structural analysis of the VP2 capsid and generating of the 2D and 3D structures was done by superimposing the Norwegian IPNV Sp strain NVI-015 (GenBank accession nos. AY379740) [20] on the template generated by Coulibaly et al. [21] (PDB Accession code 3IDE) using the SWISS MODEL workspace [22]. All manipulations aimed at determining the location of residues 217, 221 and 247 on the generated crystal structures were carried out in PyMOL v99.

### Statistical analysis

All statistical analyses were performed with the help of GraphPad Prism 5.0 (GraphPad software Inc., USA). One-way ANOVA and Student's t-test was used to calculate differences in viral replication levels and gene expression levels as indicated for each experiment. The significant level for rejection of *Ho* was set to p<0.05.

## Results

### TA and TT variants of IPNV establish persistent infection in fry

**Figure 1:**
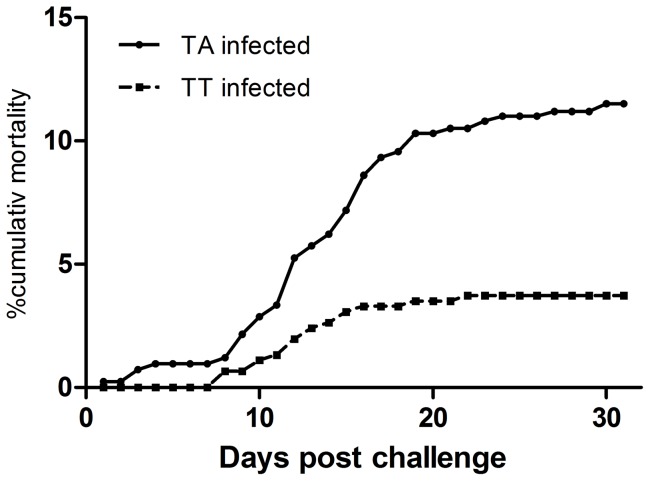
Mortality following primary challenge. The mortality in groups of naïve fry challenged with a low dose the virulent TA variant of IPNV shows a cumulative mortality of 12% while fish in the TT group has a cumulative mortality of 4%.

With the purpose to establish a persistent infection in fry and to obtain a high number of surviving fish we used a challenge dose lower than what is used in standard challenge experiments [10]. The cumulative mortality by 31 days was 12% for the TA infected fish while for TT infected fish, cumulative mortality reached 4% (Fig. 1). By one month post challenge the mortality leveled off in both groups, with a small increase by months 3 and 4 post challenge (cumulative mortalities during these 2 months were 10% in both groups). By month 5 post challenge and beyond, no fish died.

### TA and TT persistence does not result in genome variation

In order to document the infection prevalence in the two groups and persistence of infection we collected fry every month (every 30 days) from month 1–6 post infection. Five fish from each of the TA and TT infected fish were examined at each time point (n = 30, total per group) using cell culture (RTG-2 cells) and RT-PCR. Overall IPN virus was reisolated by culture and detected by RT-PCR in 29 of 30 fish in the TA group and in 30 of 30 fish examined in the TT group (Table 2). All fish in both groups were positive by culture 6 months post challenge. Fish that were found negative by cell culture were subsequently examined by RT-PCR (Table 2). These results showed that difference in mortality did not result in differences in virus prevalence for the two groups.

**Table 2:** Virus detection by cell culture and RT-PCR at persistent period (6 months).

Strains	1 month		2 months		3 months		4 months			5 months		6 months		
	CC	RT-PCR	CC	RT-PCR	CC	RT-PCR		CC	RT-PCR	CC	RT-PCR	CC	RT-PCR	
TA	5/5	Nd	3/5	2/2	0/5	4/5		2/5	3/3	2/5	3/3	5/5	Nd	
TT	5/5	Nd	4/5	0/1	0/5	5/5		2/5	3/3	1/5	4/4	5/5	Nd	

Result of reisolation by cell culture (CC) or detection of virus genome by reverse transcriptase PCR (RT-PCR). Numbers indicate positive/examined for the two methods. When all fish in one group were positive by cell culture they were not examined by RT-PCR. When fish were negative for cell culture, they were subsequently examined by RT-PCR. Strains indicate IPNV strains used for challenge and time points are time post challenge. Nd-not done.

**Figure 2:**
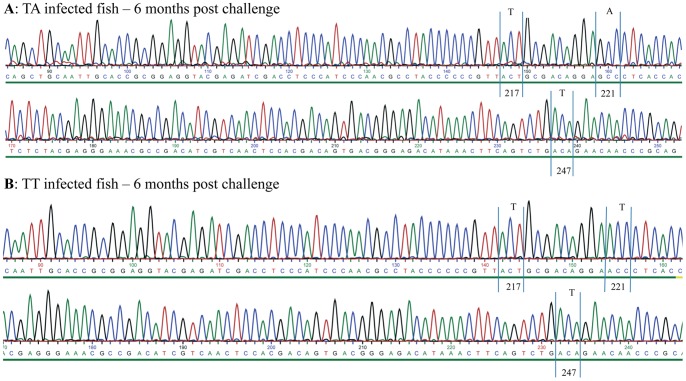
Chromatogram of TA- and TT-infected fish 6 months post challenge. Detail from chromatogram TA (A) and TT-infected (B) fish (6 months post challenge) from the hypervariable region of VP2. The chromatograms show no mutation for amino acid residues 217, 221 and 247 of VP2. Further there are no double-peaks for these positions. Details of sequencing methods are given in Materials and methods.

With the purpose to detect any mutations on the hypervariable region (HVR) of the VP2 protein (amino acid positions 180–360), RNA isolated from kidney samples from each of the five fish from each group at all sampling points was amplified by RT-PCR using primers A-Sp500F and A-Sp1689R (Table 1). PCR-products were purified by gel electrophoresis and sequenced. We found no mutation at nucleotide level on the VP2-HVRs (amino acid positions 199–319) in any of the groups up to 6 months post challenge. The results for the TA and TT group at 6 months post challenge are shown in Fig. 2A–B. Since automated sequencing methods typically select the most prevalent nucleotide we also performed a detailed analysis of the chromatograms after sequencing with the purpose to detect any double peaks. We had a particular focus on the TT infected group, since this virus strain originated from serial passages in cell culture followed by plaque purification. Again we found no complexity of the sequences of viral genomes from either of the two groups (TA- or TT-infected fish) over the persistence period, i.e. the chromatograms appear with one peak in codons encoding positions 217 and 221 of VP2 (as shown in Fig. 2A–B). We have also pinpointed position 247 of VP2 since this residue was found to vary with strain virulence in a previous study [10] and thus positions 217, 221 and 247 are indicated to show the purity of the chromatograms in TA and TT infected fish at 6 months post challenge (Fig. 2A–B).

**Figure 3:**
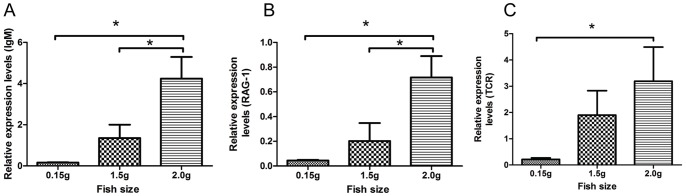
The mRNA expression of IgM, RAG-1 and TCR. The mRNA expression at different sizes of Atlantic salmon fry is indicated, showing no expression at 0.15 g size with significant increase by 1.5 g for IgM and RAG-1 while TCR is significantly upregulated by 2 g. p<0.001, n = 6; One-way Anova.

**Figure 4:**
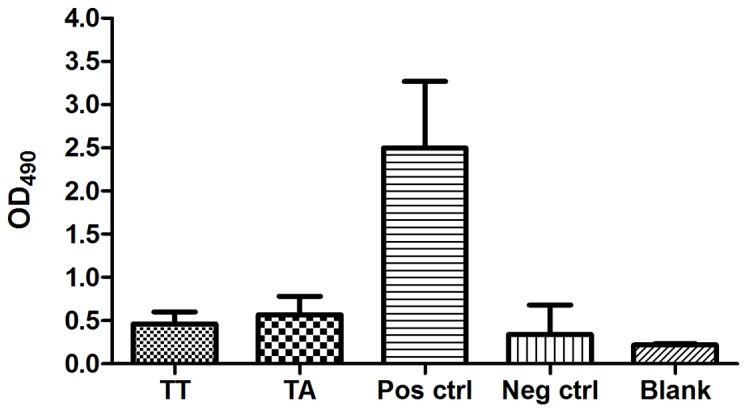
Antibody measurement in TA and TT-infected fish. ELISA results (7 months post challenge) in TA- and TT-infected fish tested against TA antigen coat. Pos ctrl are positive controls (TA) and neg ctrl are naïve fish. Blank is background without primary antibodies added. n = 3. As serum is difficult to withdraw from so small fish, we confirmed the protein concentration in the vials used and they were found comparable at 1∶50 dilution protein concentrations between 0.75 to 1.75 mg/ml. The positive control fish sample had a protein concentration around 1.6 mg/ml at the same dilution. Samples were adjusted to equal concentrations.

As there is a general understanding that immune responses will result in selection of mutated variants [23] we examined the immune status of the infected fry at time of infection (0.15 g size) by analyzing the mRNA expression of RAG-1, T cell receptor (TCR) and IgM (BCR) by real-time PCR. The finding was that all fry examined exhibited no indication of mRNA gene expression of TCR, RAG-1 or BCR at time of challenge (Fig. 3A–C) in line with the understanding that Atlantic salmon fry below 1 g are not immunocompetent. Larger fry from the same batch of fish (1.5 and 2 g) were included to document development of immunocompetence (Fig. 3A–C) and a significant increase in IgM and RAG-1 was found in 1 g fry while TCR showed significant upregulation by 2 g size (Fig. 3C). Further we also included analysis of antibody responses by a standard ELISA method to the corresponding infection strains of the virus in the two groups and found no indication of an antibody response in the persistently infected fish at end of experiment (7 months post challenge) (Fig. 4). We are thus inclined to interpret this as an indication that the fish were immunotolerant to the challenge virus.

### Stress results in down-regulation of IFN-α1 expression and increased virus replication

We hypothesized that dampening the innate responses during a persistent infection through artificially imposed stress (cortisol responses) will result in increased viral replication and thus contribute to increased diversity/mutations of the virus population. One parallel tank from each challenge group (TA or TT) was subjected to stressful treatment daily over a period of 7 days (defined as stress-period) and samplings described below were done at the first day after the stress period was over, referred to as day 1. Fish were monitored for an additional 28 days after the stress period with weekly sampling. Uninfected control fish were subjected to the same treatment while one group from each of the challenged fish and the uninfected controls were kept as non-stressed controls.

The biological effects of stress are not easily assessed in an objective manner in fingerlings of Atlantic salmon since blood samples cannot easily be withdrawn from such small fish. We therefore used infection prevalence, viral replication levels and also assessed expression of innate genes as an indication of stress. Firstly we measured virus prevalence by cell culture and RT-PCR. We collected 6 fish weekly (after the stress period) for each of the virus groups (TA and TT) and from stressed and non-stressed fish (controls) over a 28 day period. The infection prevalence had declined by 28 days in non-stressed fish irrespective of challenge strain giving 4/6 virus-positive in the TA-group (1 culture positive) and 3/6 in the TT-group (2 culture positive; Table 3). This was in contrast to the stressed groups where all fish were found virus positive by a combination of cell culture and RT-PCR, 5/6 culture positive in the TA-group and 6/6 in the TT group. The difference between virus positives in stressed and non-stressed groups is however not significant (Table 3).

**Table 3:** Virus detection by cell culture and RT-PCR post stress.

Strain/treatment	1 day post stress		14 days post stress		28 days post stress	
	CC	RT-PCR	CC	RT-PCR	CC	RT-PCR
TA/non-stressed	6/6	0	6/6	0	1/6	3/5
TA/stressed	6/6	0	6/6	0	5/6	1/1
TT/non-stressed	6/6	0	3/6	3/3	2/6	1/4
TT/stressed	5/6	1/1	4/6	2/2	6/6	0

Number of fish found positive by cell culture (cc) and reverse transcriptase PCR (RT-PCR) at different time points after the stress period was completed for the TA and TT infected groups. Fish negative by cell culture were subsequently examined by RT-PCR. Results for non-stressed controls and stressed groups are given and show that stress results in higher number of virus positive fish by cell culture at 28 days post stress. N = 6 per group.

**Figure 5:**
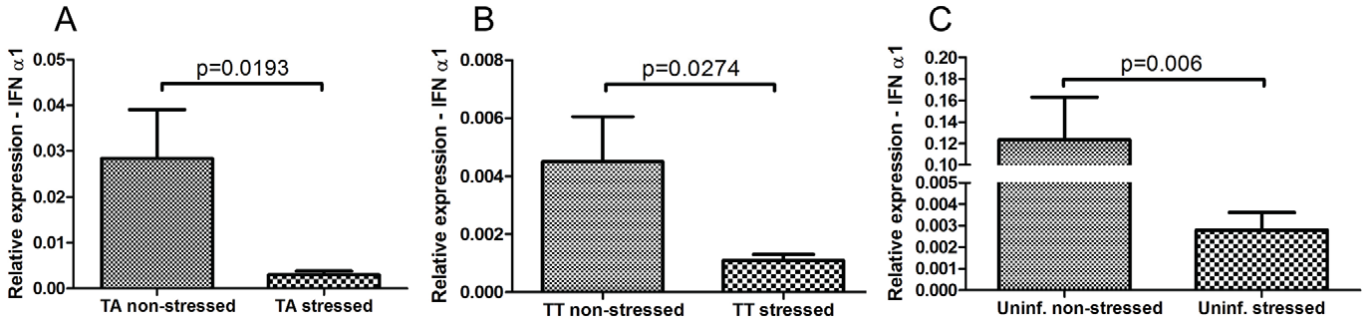
Expression of IFN-α1 mRNA in TA-, TT-infected and control fish. IFN-α1 mRNA expression in TA (A) and TT (B) infected, non-stressed and stressed fish is shown. IFNα-1 expression in uninfected fish after stress exposure is also shown (C). Stress down-regulates expression of IFN-α1 in infected fish and also in uninfected controls. P values are indicated, one-sided T-test (+SEM; n = 5 to 6 fish per group).

Virus replication was measured by real-time PCR using primers specific for segment B (Table 1) and measured as relative expression in the stressed groups relative to non-stressed controls. There was a marked increase of virus replication by Day 1 for both challenge groups, 19-fold for the TA group (p = 0.049) and 58 fold for the TT-group (p = 0.045). IFN-α1 mRNA expression were assessed by real-time RT-PCR (Table 1). We compared stressed fish to non-stressed controls for both infected and uninfected groups and we also compared the two infected groups to each other, all at Day 1. Six fish were analyzed in each group (TA, TT, and uninfected) and for each treatment, stressed and non-stressed (36 fish total). In the stressed fish, down-regulation of IFN-α1 mRNA expression was significant for both TA (Fig. 5A) and TT-infected groups (Fig. 5B) as well as for the uninfected fish (Fig. 5C).

### Reversion to virulence occurs at the end of the stress period

**Figure 6:**
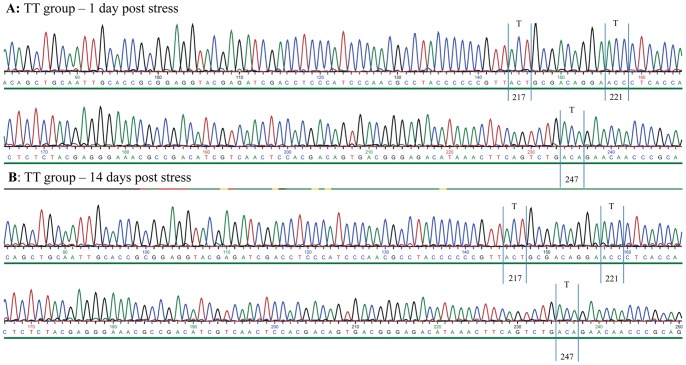
Chromatogram of TT-infected fish 1 and 14 days post stress. Details from chromatogram of TT-infected fish at 1 day (A) and 14 days (B) post stress. Area indicated is from the hypervariable region of VP2. The chromatograms show that amino acid positions 217, 221 and 247 remain unchanged. Further there are no double-peaks in codons of any of these residues.

**Figure 7:**
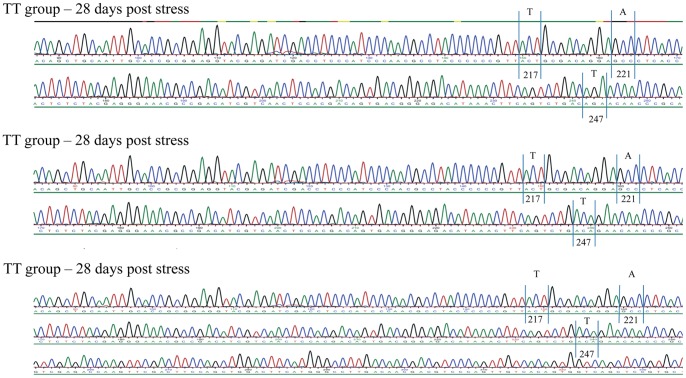
Chromatograms for TT-reverted fish at 28 days post stress. Details from chromatogram of three TT-infected fish at 28 days post stress. Codons encoding amino acid residues 217, 221 and 247 are detailed and show reversion at amino acid residue 221. Noticeably, there are no double-peaks in codons of any of these residues.

Re-isolation of virus and genome variation was assessed by sequencing re-isolated virus or PCR products obtained directly from kidney from 6 fish from each challenge group (TA and TT) at days 1, 14 and 28 post stress in stressed and non-stressed groups (24 fish total). For the TT-group we found no changes by day 1 or day 14 (24 fish, non-stressed and stressed examined in total at these time points, Fig. 6A–B). By Day 28 there was a non-synonymous mutation in the stressed group in 3 out of 6 fish giving a T221A (VP2) mutation for all three fish (Fig. 7A–C). This resulted in a motif typical of a virulent T_217_A_221_ variant of IPNV. In the non-stressed group TT-infected fish, no mutation was found by Day 28 (Fig. S1). In the TA-infected group (12 fish examined), in both stressed and non-stressed individuals, no mutation was found.

**Figure 8:**
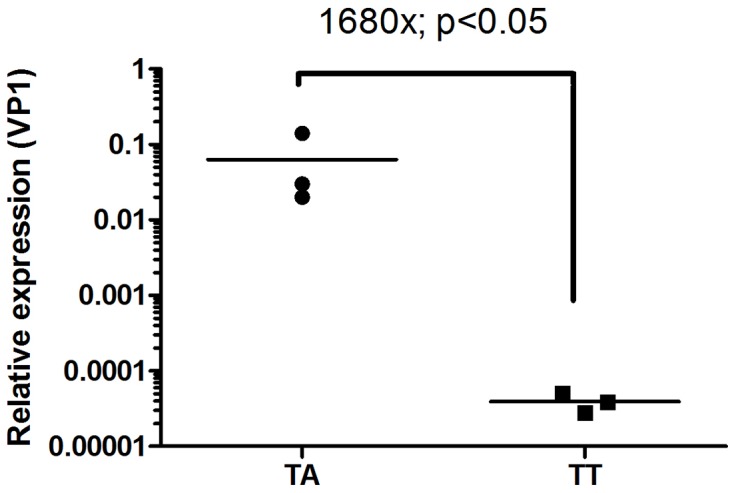
Viral replication in TA-reverted and non-reverted TT infected fish. Relative viral expression at 28 days post stress in three TA-reverted versus three non-reverted fish of the group originally infected with the TT-strain. Relative replication levels in the TA-reverted fish are 1680-times higher than in the TT-individuals. P-value, one-sided T-test; n = 3, log_2_ values.

An interesting finding was that the chromatograms for the 3 mutated strains (T221A) were found without any double peaks or mixes of nucleotides in the positions encoding residue 221 of VP2 (Fig. 7A–C). This was unexpected and it motivated us to compare the virus replication level in kidney samples of reverted and non-reverted fish of the TT group by isolating viral RNA directly from tissue specimens. We examined virus replication by estimating the expression of segment B of IPNV (encoding VP1; as described above) by real-time PCR and we found a 1680-fold up-regulation of virus load by day 28 in TA-reverted relative to the TT-non reverted fish (Fig. 8) pointing towards highly contrasting replication capacities for the two strains. We therefore consider it likely that the “purity” and lack of double-peaks of the chromatograms for the reverted isolates is due to the clonal expansion of the T221A variants which results in an exclusion of the TT variant.

### TA strains are less sensitive to an IFN-α1 induced antiviral state than TT variants in vitro

**Figure 9:**
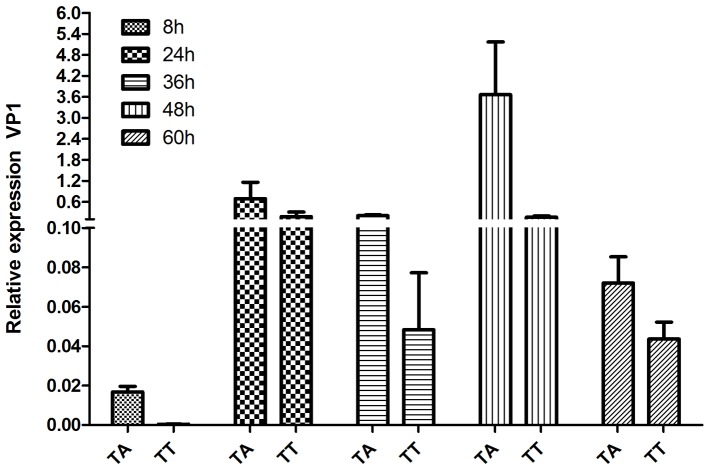
Recombinant IFN-α1 effect on viral replication of TA and TT infected TO cells. Viral replication levels (VP1 expression) at different time post infection (hours post infection; hpi) of TO cells, pretreated (24 h) with recombinant IFN-α1. The TA strain shows earlier increase of replication levels (24 hpi) and peak at 48 hpi at 23-fold higher replication levels than the TT-strain at this time point. N = 3–4 replicates per time point.

The findings reported above are indicative of the TA-variant replicate to higher levels which results in an exclusion of the lesser fit TT-variant of IPNV. Further, in light of recent publications showing that IPNV has the ability to reduce/block IFNα-induced responses in vitro [24], [25] we examined replication differences between TA and TT strains under IFNα-1 influence. We therefore performed an in vitro study with the purpose to explore the possibility that TA and TT strains differ in their sensitivity to IFN-α1 induced antiviral responses. This could possibly shed light on the differences observed *in vivo*. We did this by comparing replication levels of TA and TT strains in macrophage cell line from salmon (TO cells; [15]). We induced an antiviral state in the cells by pretreating them with recombinant IFN-α1 [26] 24 hours prior to infection also including untreated parallels. Interestingly, we found that the TA strain had 23-fold higher replication level than the TT strain at peak expression time (48 h post infection; Fig. 9) indicating a difference in sensitivity to the antiviral state induced by rIFN-α1 treatment. It should be noted that the replication of both virus strains was markedly reduced compared to untreated controls (15-20) fold lower for both strains compared to non-IFNα-1 treated cells, not shown).

### Reverted T221A variants are virulent to fry of salmon

**Figure 10:**
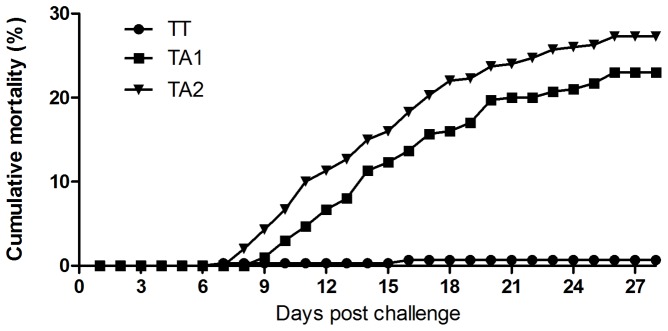
Cumulative mortality induced by reverted virus strains. The cumulative mortality in the fish groups challenged with the TA-reverted strains reached close to 30% while the TT-challenged fish were not different from non-infected controls. Mortality leveled off at day 26 post challenge. Three parallel tanks for each challenge group, plus non-challenged controls (not shown).

Since no mortality was observed in the TA-reverted fish post stress we decided to determine if the reverted strains were virulent to susceptible fry of Atlantic salmon. The three virus strains isolated from TA-reverted fish (originally infected with the TT strain) were used to challenge fry at time of start feeding and a parallel group of fry was challenged with the TT isolate. The cumulative mortality at end of challenge was close to 30% and 0.7% for the TA- and TT-infected fish, respectively (Fig. 10), showing that the reverted strains had recovered their virulent traits. The third TA strain gave lower mortality (average 9%) because of lower titer in the challenge inoculum (as a result of a too high dilution was used at time of challenge). IPNV was also re-isolated from challenged fish and their genome amplified by RT-PCR for sequencing (segment A). The obtained sequences from re-isolated virus were identical to the isolates used for challenge, both for TA and TT strains (not shown).

### Structural analysis of the VP2-HVRs

**Figure 11:**
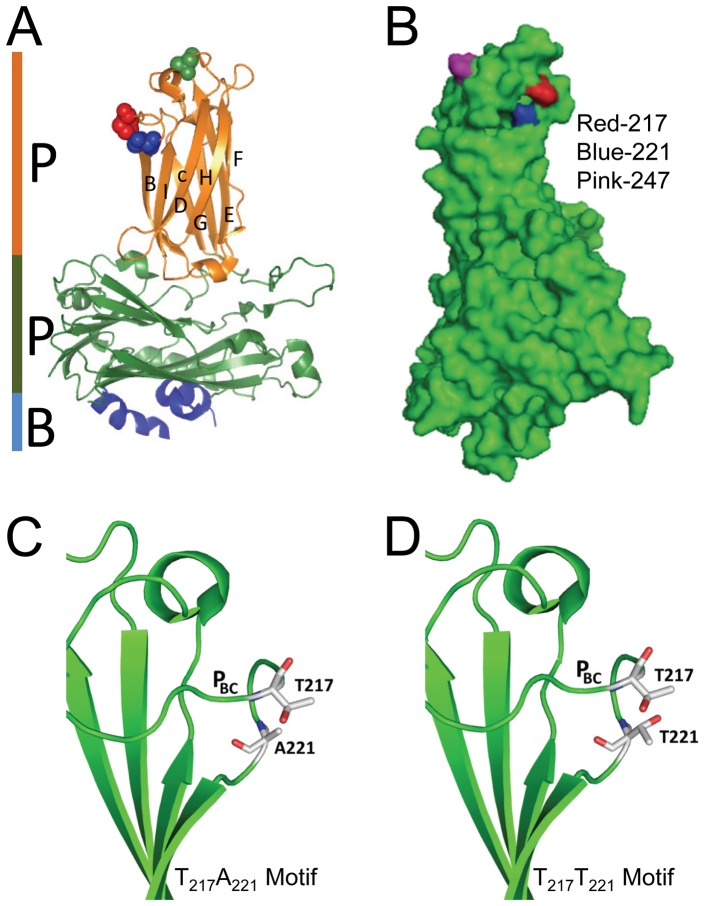
Crystal structure of the VP2 subviral particle (SVP). **A**) Shows the P, S and B domains of the VP2-SVP while spheres represent locations of residues 217 (red), 221 (blue) and 247 (green). Note that all three residues are located on the VP2-HVRs of the P-domain. Residues 217 and A221 are close to each other on loop P_BC_ which is the outermost loop of the VP2-HVRs while T247 is on the apex of loop P_DE_. **B**) Displays positions 217, 221 and 247 on the 3D SVP. Note that positions 217 and 221 are next to each other. **C**) 2D cartoon of the T_217_A_221_ motif with an alanine at 221 projecting its hydrogen bond (red) inwardly towards the inner core of the capsid protein (green) while the non-reactive end projects outwardly towards T217. **D**) 2D cartoon of the T_217_T_221_ motif. Note the curving of loopP_BC_ bringing positions 217 and 221 close to each other. Note also the two threonines projecting out their hydrogen bonds (red) outwardly and are proximal to each other.

Positions 217, 221 and 247 were associated with virulence in field strains of IPNV in a previous study [10] and pinned down to residues 217 and 221 using reverse genetics [11]. To gain a better understanding on the structural properties influencing virulence of Sp strain NVI015, we analyzed the structural layout and impact of mutational changes on residues influencing the virulence motif. Structural analysis of the VP2 subviral particle (SVP) shows that residue 217 and 221 are located on loop P_BC_ on the P-domain of the hypervariable region (HVR) of the VP2 capsid (Figure 11A). Although 217 and 221 are four residues apart, the structural fold on the backbone molecule brings these residues in close proximity as shown on the 3D structure that these residues are located next to each other (Figure 11B). This suggest that these residues complement each other's activity making them function as a single motif. Structural layout of individual residues shows that threonine both at 217 and 221 projects outwardly giving its hydrogen bonds for potential binding to cell receptors. On the contrary, an alanine at 221 projects its hydrogen bond inwardly towards the inner core of the capsid protein leaving the non-reactive end to project towards the outer surface of the protein capsid (Figure 11C). Put together, two threonines at 217 and 221 having their hydrogen bonds projecting outwardly towards each other (Figure 11D) suggests that the T_217_T_221_ motif has a higher binding potential than the T_217_A_221_ motif that has an alanine at 221 (Figure 11C). Hence, a T221A mutation suggests that it could influence the hydrophobicity property of the virulence motif.

## Discussion

In this study we show that attenuated IPNV virus strains establish a persistent infection and revert to virulence following stress exposure of Atlantic salmon fry. Stress lowers the expression of IFNα, which seem to reduce the anti-viral state and allows for increased replication levels of IPNV. The reverted variant, T_217_A_221_, has superior replication capacity which leads to a purifying selection and a complete dominance over the T_217_T_221_ variant *in vivo*. Concordant with *in vivo* findings, the T_217_A_221_ variant replicates under high levels of IFNα in vitro and was partly resistant to rIFN-α1 induced antiviral responses. This could provide an explanation for what appears as a competitive exclusion *in vivo*.

As pointed out by Betts and Russell [27] that threonines are quite common on protein functional sites and it is likely that the strategic location of threonine at 217 and 221 is indicative of its functional involvement in binding to host cell receptors. Besides, threonine has been reported to have variable binding abilities and intracellular threonines can be phosphorylated while in extracellular environments they can be O-glycosylated enabling their attachment to different receptors [27]. On the other hand, it has been shown that alanine is a less reactive residue with its side chains being less likely to attach to cell receptors and has the tendency to project inwardly towards the inner core of the protein [27], [28] which is in line with our observation on A221 (Fig. 11). Put together, these observations suggest that the T_217_T_221_ motif is likely to have a higher binding ability than T_217_A_221_ to cell receptors. However, how the apparently weaker T_217_A_221_ with less binding potential is translated into higher replication capacity, 1680 times higher than the T_217_T_221_, is not clear. Bauer et al [29] reported a similar observation that substituting an alanine for valine at position 296 on the VP1 capsid of polyomavirus reduced the binding avidity of the virus. Site directed mutagenesis showed that introducing A296 showed full virulence while V296 that had a higher binding avidity and reduced virulence [29], [30]. In their observation, Bauer et al [29] noted that too strong an avidity for a receptor could inhibit virus escape from infected cells resulting in less efficient release of virus from bound receptors. To enhance virus release, viruses like influenza depend on enzymes like neuraminidase which is a receptor-destroying-enzyme used to aid virus release and facilitate its spread to other cells as a way of enhancing virulence [31], [32]. Therefore, small changes in receptor avidity, for viruses such as polyomavirus and IPNV that do not have receptor-destroying enzymes could have a significant effect on virus release from bound cell-receptors. Hence, in the present study a T_217_T_221_ motif with high binding potential entails reduced efficiency in the release of virus bound to cell receptors subsequently leading to persistent infections due to failure or slow release of virus. This phenomenon supports our earlier findings in which we documented that a threonine at 221 was linked to IPNV persistent infections [11]. On the contrary, it is likely that a mutation from a threonine to an alanine, which is less reactive, at 221 reduces the binding avidity leading to efficient release of the virus which may enhance its spread and increase in replication capacity. Hence, stress induced mutation from T221A could have reduced the strong binding avidity of the T217T221 motif to a low binding motif. Put together, the high replication capacity and ability of the reverted T_217_A_221_ variant to produce higher mortality (30%) than the T_217_T_221_ variants (0.7%), could explain factors leading to recrudescence from persistent infections to active infections seen under field conditions. IPN is often stress associated especially in postmolts in which outbreaks occur soon after transfer to seawater [7], [33]. However, there is need for detailed studies aimed at identifying and characterizing the IPNV cell-receptor in order to enhance our understanding of the process of virus entry and release, and to fully elucidate how these polymorphisms play an important role in disease progression.

Yet another aspect is the possibility that an alanine at position 221 represents improved translation efficiency developed as an adaptation to the host. It is known that codon usage can impact translation efficiency and this is seen particularly for proteins that appear abundantly in the virion, typically structural proteins [34]. Translation efficiency can also relate to GC content of the host genome and RNA folding processes [35], [36]. The possibility of the tRNA population is biased towards tRNA-Ala over tRNA-Thr should also be considered but to the knowledge of the authors, little is known about these factors in Atlantic salmon.

No mutation was found by sequencing in persistently infected fish over a period of 6 months. The initial challenge was performed in fry at a physiological state where no recombination of TCR or BCR genes had occurred, *i.e.* infection was carried out prior to immunocompetence had developed and in line with these findings no antibody responses were detected in persistently infected fish at 7 months post challenge. The general understanding is that persistence typically occurs as a result of changes in antigenic epitopes induced by adaptive immune responses while in the absence of immune pressure there should be less variation [37]–[39]. The observed lack of genomic variability for both TA and TT infected groups concurs with the observation that infected fish had no circulating antibodies, i.e. lack of selective pressure. Our interpretation has been that fish are immunotolerant to the virus as suggested earlier for IPNV infected fish [40]. However immunotolerance is a highly specific, adaptive immune response and innate responses will still contribute to keep the virus replication in check. Hanada and coworkers [41] discuss the possibility that replication frequency contribute more importantly to genetic variability than replication error per se since the error rate is more or less constant for most viruses. The observation that the TT variant revert to wild-type after stress-induced modulation of the innate immune responses is interesting, particularly since this can be linked to increased replication frequency.

Another factor that possibly contributes to the dominance of TA over TT is the lesser sensitivity to IFN-α1 induced antiviral responses. The inhibitory activity of Mx on IPNV replication has been documented earlier [42] however differences between strains of IPNV were not explored. Here we showed that the replication capacity of the TA strain was higher than the TT variant, peaking at 23-fold higher by 48 h post infection still much lower than what was seen for cell cultures not pretreated with IFNα-1.

The T_217_T_221_ strain was made by serial passage in cell culture (CHSE) resulting in mutation at position 221 of VP2 followed by plaque purification [11], [27]. In vivo stress caused increased virus replication and mutation to T_217_A_221_. Reversion to wild-type is a known phenomenon for many viruses. In our study one cannot exclude the possibility that the plaque purified variant used for challenge of the TT group also contained a minority of clones of a TA variant and thus the TA found in the stressed TT group would merely represent a purifying selection of clones that were already present at the time of challenge. We do however consider this less likely based on the fact that the persistent infection was established through challenge of naïve fish. The general experience is that experimental challenge of susceptible fry will favor the most fit variant and thus it is highly likely that a TA variant present at low number in for example a TT biased population would take dominance over the less fit variant. Further the fish were followed over a period of 6 months prior to stress-exposure and not one single isolate sequenced in the TT group showed any indication of nucleotide diversity at the codon encoding residue 221 of VP2. Sequencing of PCR products represents a selection step where the more prevalent variants would be favored. However, it is currently a standard method used for sequencing of viruses, particularly when combined with examination of chromatograms [23].

Concluding, we show here for the first time that attenuated virus variants of IPNV can revert to virulence *in vivo* and that stress plays a key role in facilitating increased viral replication with subsequent mutation and reversion to a virulent form.

## Supporting Information

Figure S1 Chromatograms for TT infected, non-stressed groups. Non-stressed fish originally infected with the TT strain showed no mutation in position encoding residue 221 of VP2. Example from two individual fish examined at this time point.
(TIF)
