# Supplementary figures and images for: Stress-Induced Reversion to Virulence of Infectious Pancreatic Necrosis Virus in Naïve Fry of Atlantic Salmon (Salmo salar L.)
Source: PLoS One. 2013 Feb 19;8(2):e54656. doi: 10.1371/journal.pone.0054656 (PMC3576400; doi:10.1371/journal.pone.0054656)

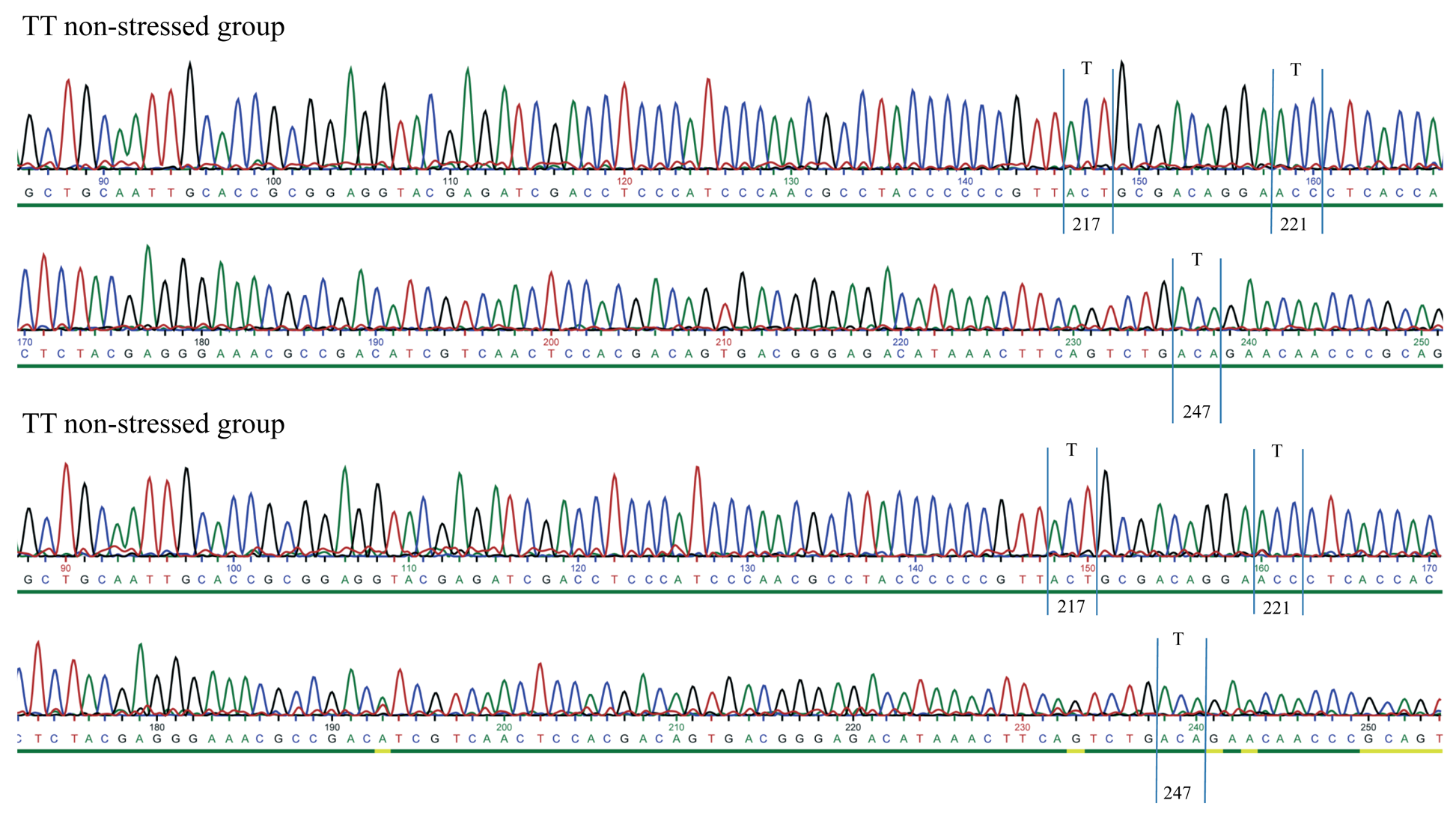

Supplement: Figure S1 — Chromatograms for TT infected, non-stressed groups. Non-stressed fish originally infected with the TT strain showed no mutation in position encoding residue 221 of VP2. Example from two individual fish examined at this time point. (TIF) [file pone.0054656.s001.tif]
